# Adaptive Dynamic Programming-Based Multi-Sensor Scheduling for Collaborative Target Tracking in Energy Harvesting Wireless Sensor Networks

**DOI:** 10.3390/s18124090

**Published:** 2018-11-22

**Authors:** Fen Liu, Wendong Xiao, Shuai Chen, Chengpeng Jiang

**Affiliations:** 1 School of Automation and Electrical Engineering, University of Science and Technology Beijing, Beijing100083, China; liufenustb@163.com (F.L.); shuai19860230@163.com (S.C.); jcp09868@163.com (C.J.); 2 Beijing Engineering Research Center of Industrial Spectrum Imaging, Beijing 100083, China

**Keywords:** energy harvesting, wireless sensor networks, adaptive dynamic programming, target tracking, extended Kalman filter, sensor scheduling

## Abstract

Collaborative target tracking is one of the most important applications of wireless sensor networks (WSNs), in which the network must rely on sensor scheduling to balance the tracking accuracy and energy consumption, due to the limited network resources for sensing, communication, and computation. With the recent development of energy acquisition technologies, the building of WSNs based on energy harvesting has become possible to overcome the limitation of battery energy in WSNs, where theoretically the lifetime of the network could be extended to infinite. However, energy-harvesting WSNs pose new technical challenges for collaborative target tracking on how to schedule sensors over the infinite horizon under the restriction on limited sensor energy harvesting capabilities. In this paper, we propose a novel adaptive dynamic programming (ADP)-based multi-sensor scheduling algorithm (ADP-MSS) for collaborative target tracking for energy-harvesting WSNs. ADP-MSS can schedule multiple sensors for each time step over an infinite horizon to achieve high tracking accuracy, based on the extended Kalman filter (EKF) for target state prediction and estimation. Theoretical analysis shows the optimality of ADP-MSS, and simulation results demonstrate its superior tracking accuracy compared with an ADP-based single-sensor scheduling scheme and a simulated-annealing based multi-sensor scheduling scheme.

## 1. Introduction

A wireless sensor network (WSN) is usually deployed to monitor the physical phenomena in the geographic area covered by a large number of sensor nodes. It has the advantages of low cost, rapid deployment, self-organization, and fault tolerance, with wide applications such as environmental monitoring [1,2], medical care [3], pension service [4], and intelligent transportation [5].

Target tracking is a typical research problem in WSNs for studying collaborative signal and information processing where the sensors are scheduled by considering the tracking performance and the constrained network resources (e.g., the wireless bandwidth and limited sensor energy).

Usually, sensor scheduling relates to tasking the appropriate sensors at the right time to achieve satisfactory performance by considering the limited sensing, computing, and communication resources of the sensors. Effective sensor scheduling algorithms are needed for collaborative target tracking in order to get accurate estimates and effective resource utilization. For example, sensors are scheduled by using dynamic clusters and duty cycling technology to economize the limited energy of the sensors in [6]. An adaptive sensor scheduling scheme was proposed in [7] to improve tracking accuracy on the premise of employing the same number of sensors based on extended Kalman filter (EKF). However, these existing sensor scheduling methods are based on the local optimization of performance for limited time steps, and may be sub-optimal from a global perspective (especially regarding the performance for all the time steps from the current one to the end or infinity).

Additionally, with the development of new technologies such as new materials, microelectronics, energy storage, and conversion, energy harvesting technologies that can obtain energy from a variety of energy sources in the environment (e.g., light [8], wind [9], thermoelectric energy [10,11], electromagnetic radiation [12]) have recently been developed. Energy harvesting can be used to charge batteries from time to time, and can help to avoid battery replacement. Energy harvesting technologies make it possible for the sensor nodes to obtain energy from the environment at a low cost. Accordingly, an energy harvesting-based WSN was introduced in which the sensor nodes could harvest energy from the environment to power the sensors [13].

Theoretically, the lifetime of an energy harvesting-enabled network is infinite. Subsequently, the key problem for sensor scheduling is no longer to maximize the network lifetime, but to optimize the network performance under the given energy harvesting capability. Hence, the development of energy harvesting technologies has provided a new challenge of infinite-horizon sensor scheduling with finite energy harvesting capability for high-performance target tracking. In this paper, we apply adaptive dynamic programming (ADP) to sensor scheduling for collaborative target tracking in an energy-harvesting WSN over an infinite horizon.

ADP was proposed by Werbos [14]. The typical structure of ADP consists of a critic module, a state transition module, and an action module. Each module can be realized by a neural network (NN) [15,16,17]. Characterized by strong abilities of self-learning and adaptation, ADP has demonstrated a strong capability to find the optimal control policy and solve the discrete system dynamic programming (DP) problem [18], including the adaptive critic design, reinforcement learning [19], and so on, obtaining the approximate optimal performance and the optimal control to satisfy the Bellman optimal principle through the function approximation structure. An ADP-based sensor scheduling scheme for target tracking in an energy-harvesting WSN was proposed in [20], which made the sensor energy consumption and tracking accuracy optimal over the system operational horizon for WSNs. However, only one sensor was scheduled for each time step, and therefore the tracking accuracy improvement was limited. In contrast, due to the energy harvesting capabilities, the motivation of this paper is to present a novel multi-sensor scheduling scheme for global performance optimization over an infinite horizon.

The rest of this paper is organized as follows: In Section 2, the related work is introduced. In Section 3, the energy harvesting model is established based on solar energy harvesting, and the EKF-based target motion model and measurement model are introduced. In Section 4, the details of the target tracking system and the predicted energy consumption model are given. In Section 5, the optimal multi-sensor scheduling problem is abstracted to a mathematical model and the ADP-based multi-sensor scheduling algorithm (ADP-MSS) is proposed. In Section 6, the performance of the proposed algorithm is examined by theoretical analysis and simulations. In Section 7, conclusions are drawn and some suggested future work is discussed.

## 2. Related Work

In the existing work for target tracking in WSNs, various sensor scheduling schemes have been proposed. By considering the number of scheduled sensors for each time step, they can be classified into one-sensor scheduling schemes and multi-sensor scheduling schemes. Meanwhile, by considering the scheduling mechanisms, they can be classified into non-adaptive sensor scheduling schemes and adaptive sensor scheduling schemes.

For example, a periodic sensor scheduling (PSS) scheme in which sensors sense the target alternatively within the predefined time slots was presented to avoid the inter-sensor interference (ISI) problem and utilize the sensors more effectively [21]. The drawback of PSS is the existence of an empty detection when a scheduled sensor cannot generate an effective measurement, which results in lower tracking accuracy and the wasting of sensor power. A distributed sensor scheduling scheme was proposed in [22] where the tasking sensor is elected spontaneously from the sensors with pending sensing tasks via random competition based on carrier sense multiple access. Each node does not need to know the location of the other nodes, which requires less occupied memory. However, the computation burden of the scheduling is shared to all the active nodes. Aiming to optimize the tradeoff between the tracking accuracy and the energy cost for collaborative target tracking in WSNs, a dynamic sensor selection scheme based on genetic algorithms was proposed in [23].

In the above approaches, only one sensor node is scheduled for performing the measurement at each time step. Generally, tracking performance can be further improved by multi-sensor scheduling. For example, a distributed multi-sensor target tracking algorithm was proposed in [24] by using a cluster-based Kalman filter (KF). At each time step, one sensor is selected as the head to fuse the measurements from the other sensors, estimate the target state using EKF, and send the results to the base station. A distributed-saturation-degree-based algorithm was proposed for target tracking with multiple ultrasonic sensors, where the ISI avoidance problem is converted to the problem of multiple access in a shared channel and the scheduling problem is transformed to a coloring problem [25]. In [26], probability-based prediction and sleep scheduling protocols were presented to improve energy efficiency with limited tracking performance loss. The above work does not analyze the energy consumption of the sensor node, and lacks the adaptation mechanism in response to energy changes in the network.

Some adaptive sensor scheduling solutions can be found in the literature. For example, an adaptive sensor scheduling scheme was introduced by scheduling the next tasking sensor for the next time step according to the predicted tracking accuracy derived from the trace of the covariance matrix of the state estimation [21]. In [27], an energy-efficient target tracking method was proposed, where the KF is used to predict the target location for the next time step, then the sensor node and the cluster are selected to minimize the energy consumption. A multi-step sensor scheduling scheme is adopted based on the adaptive sampling interval approach to achieve fast tracking speed and superior energy efficiency without degrading the tracking accuracy [28]. To improve the performance of energy efficiency and tracking accuracy, authors in [29] proposed a multi-step sensor scheduling approach using the branch-and-bound algorithm. It could achieve the optimal multi-step sensor scheduling solution, but easy led to the “curse of dimensionality” problem.

Nevertheless, the above adaptive sensor scheduling solutions only choose one sensor node for each time step. Similarly, a flexible mechanism to improve the tracking performance can be obtained by adaptive multi-sensor scheduling. For example, in [30], a distributed adaptive multi-sensor scheduling was presented to implement the target tracking with the cooperation of the sensor nodes. In the adaptive sampling interval approach for single target tracking, the sensors are scheduled in alternative tracking mode to implement energy-efficient tracking according to the predicted tracking accuracy based on EKF [31]. To minimize the estimation error over multiple time steps in a computationally tractable fashion, Huber [32] proposed an information-based pruning algorithm for multi-step sensor scheduling by using the information matrices of the sensors and the monotonicity of the Riccati equation. In [33], several suboptimal scheduling algorithms were proposed with the performance expressed by the weighted sum of the estimated error covariance matrix in KF and the energy consumption. The posterior Cramer–Rao lower bound was proposed as a sensor selection metric, which put a constraint on the total number of selected sensors to observe the target over a time window [7,34].

However, all these methods dispatch the sensors based on the optimization of local performance, instead of global performance. In energy-harvesting WSNs, novel design criteria are required to achieve an overall performance optimization over an infinite horizon.

There are some studies in the literature on energy harvesting-based WSNs. For example, the Markov decision process (MDP) was presented to maximize the long-term expected throughput to derive the optimal power level [35]. However, the computational complexity of the MDP-based approaches is generally high due to the large volume of the state and action space. To optimize the transmission performance, Lyapunov optimization theory was used in an energy harvesting wireless communication system [36]. DP was proposed for the optimization of the task scheduling to maximize the quality of service in a solar energy-harvesting Internet of Things [37]. However, DP easily leads to the “curse of dimensionality”.

Recently, data-driven approaches have been widely used in the control field to realize a variety of data-based linear and nonlinear systems, for prediction, evaluation, scheduling, monitoring, diagnosis, decision-making, and optimization [38,39,40,41,42,43,44,45,46]. ADP is a typical data-driven approach for control over an infinite horizon, which can avoid the “curse of dimensionality” problem and the reverse solving problem existing in DP [47]. Up to now, ADP has been applied to nonlinear zero-/nonzero-sum differential games [48,49], optimal tracking control problems [50], optimal control of intelligent grid [51,52], and optimal time slot scheduling of MAC protocol [53]. Recently ADP was also proposed as an optimal sensor scheduling scheme for target tracking in an energy-harvesting WSN [20], by scheduling one sensor for each time step over an infinite horizon considering the global tracking accuracy and energy consumption. However, ADP-based multi-sensor scheduling for collaborative target tracking in energy-harvesting WSNs remains as an open and challenging problem.

## 3. Basic Models

A sensor node usually consists of a sensing unit, a processing unit, a transceiver unit, and a power unit. The energy is finite in a power unit, while the energy of an energy-harvesting sensor node can be collected by the energy harvesting device through ambient energy from time to time and stored as electric energy.

In this paper, we assume that solar energy-based harvesting technology is adopted by the sensor nodes. We also assume that the WSN of this paper is composed of one sink node and *M* energy-harvesting sensor nodes. Each sensor node with enough energy can sense the target in its sensing region and transmit the perceived information to the sink node. The sink node fuses the received measurements, predicts the target states, and performs sensor scheduling.

### 3.1. Solar Energy Harvesting Model of the Sensor Nodes

In this paper, we assume that solar energy harvesting is used by the sensor.

If the sensor’s energy storage capacity is unlimited, the harvested energy of a sensor node can be modeled as [54]:

(1) $$E_{h}(t_{0},\Delta T)=\int_{t_{0}}^{t_{0}+\Delta T}\eta_{e}f_{e}(t)dt=\eta_{e}\int_{t_{0}}^{t_{0}+\Delta T}f_{e}(t)dt,$$

where $t_{0}$ is the starting time for energy harvesting, ΔT is the time duration, $f_{e}(t)$ is the statistical distribution of the solar energy, and $\eta_{e}$ is the conversion efficiency of the solar panel.

However, the unlimited storage capacity is impractical. Suppose that the maximal energy storage capacity of sensor i is $H_{i}^{\max}(0<H_{i}^{\max}<\infty )$. Then, the harvested energy of sensor i with the residual energy $E_{left}$ is $\min\left(E_{left}+E_{h}(t_{0},\Delta T),H_{i}^{\max}\right)$.

### 3.2. EKF-Based Prediction and Estimation Model for Target State

In this paper, we will apply EKF to the target tracking problem. The basic idea is to use minimum mean square error as the best estimation criterion and update the current estimated state with the previous prediction and the current measurements [55]. In this paper, we adopt a linear target motion model and a non-linear measurement model, both with Gaussian noise distributions.

The state of the target at the *k*-th time step at $t_{k}$ is

(2) $$X(k)={\left[\begin{array}{cc} \begin{array}{cc} x(k) & v_{x}(k) \end{array} & \begin{array}{cc} y(k) & v_{y}(k) \end{array} \end{array}\right]}^{T},$$

where (x(k), y(k)) are the location coordinates of the target and $\left(v_{x}(k),v_{y}(k)\right)$ are the velocity of the target along the *x*-axis and the *y*-axis at $t_{k}$. The target motion is modeled by the following constant velocity motion model

(3) $$X(k+1)=A(\Delta t_{k})X(k)+w(\Delta t_{k}),$$

(4) $$A(\Delta t_{k})=\left[\begin{array}{cccc} 1 & \Delta t_{k} & 0 & 0\\ 0 & 1 & 0 & 0\\ 0 & 0 & 1 & \Delta t_{k}\\ 0 & 0 & 0 & 1 \end{array}\right],\;Q(\Delta t_{k})=q[\begin{array}{cccc} \frac{\Delta t_{k}^{3}}{3} & \frac{\Delta t_{k}^{2}}{2} & 0 & 0\\ \frac{\Delta t_{k}^{2}}{2} & \Delta t_{k} & 0 & 0\\ 0 & 0 & \frac{\Delta t_{k}^{3}}{3} & \frac{\Delta t_{k}^{2}}{2}\\ 0 & 0 & \frac{\Delta t_{k}^{2}}{2} & \Delta t_{k} \end{array}].$$

If the target is detected by *n* sensors, then the sink will obtain *n* measurements $z_{j}(k)\left(j=1,\cdots ,n\right)$. Let $Z(k)={\left[z_{1}(k),z_{2}(k),\cdots ,z_{n}(k)\right]}^{T}$, then the measurement model is given by

(5) $$Z(k)=\left[\begin{array}{c} h_{1}\left(X(k)\right)\\ h_{2}\left(X(k)\right)\\ \vdots \\ h_{n}\left(X(k)\right) \end{array}\right]+\left[\begin{array}{c} v_{1}(k)\\ v_{2}(k)\\ \vdots \\ v_{n}(k) \end{array}\right]=h\left(X(k)\right)+v(k).$$

Some notations used in EKF are listed as follows:

$\hat{X}(k+1|k)$: Step prediction for the (*k* + 1)-th time step using state estimation at the *k*-th time step.
$\hat{X}(k+1|k+1)$: State estimation at the (*k* + 1)-th time step.
$\Delta t_{k}$: Sampling time interval between two successive time steps.
$w(\Delta t_{k})$: Process noise at the *k*-th time step.
$Q(\Delta t_{k})$: Covariance matrix of the process noise at the *k*-th time step.
q: Given scalar that represents the intensity of the process noise.
$v_{i}(k)$: Measurement noise of sensor *i* at the *k*-th time step.
v(k): Measurement noise at the *k*-th time step.
$R_{i}(k)$, R(k): Covariance matrix of the measurement noise at the *k*-th time step.
$h_{i}\left(X(k)\right)$, h(X(k)): Measurement function at the *k*-th time step.
H(k+1): Jacobian matrix of *h* at $t_{k+1}$ with respect to $\hat{X}(k+1|k)$.
P(k+1|k): Error covariance matrix of the state prediction for the (*k* + 1)-th time step.
P(k+1|k+1): Error covariance matrix of the state estimation at the (*k* + 1)-th time step.
I: Unit matrix.
K(k+1): Kalman gain at the (*k* + 1)-th time step.

Both $w(\Delta t_{k})$ and $v_{i}$ are independent and assumed to have zero-mean, white, Gaussian probability distributions. $h_{i}$ is generally non-linear depending on X(k), the measurement characteristic, and the parameters (e.g., the location) of sensor *i*.

In EKF, the prediction is operated as

(6) $$\hat{X}(k+1|k)=A(\Delta t_{k})\hat{X}(k|k),$$

(7) $$P(k+1|k)=A(\Delta t_{k})P(k|k)A^{T}(\Delta t_{k})+Q(\Delta t_{k}).$$

The estimation is operated as

(8) $$K(k+1)=P(k+1|k)H^{T}(k+1){\left[H(k+1)P(k+1|k)H^{T}(k+1)+R(k+1)\right]}^{-1},$$

(9) $$\hat{X}(k+1|k+1)=\hat{X}(k+1|k)+K(k+1)\left(Z(k+1)-h\left(\hat{X}(k+1|k)\right)\right),$$

(10) P(k+1|k+1)=(I−K(k+1)H(k+1))P(k+1|k).

### 3.3. Tracking Accuracy

At the *k*-th step, the sink node schedules the sensors to minimize the global performance, which is composed of the energy consumption and tracking accuracy, under the limited energy harvesting capabilities. However, it is impractical to calculate the error through the difference between the real state and the estimated state because the measurement is unobtainable prior to the sink scheduling the sensors. However, the error covariance based on EKF is available before measuring, and it describes the degree of the difference between the estimation and the expectation. Hence, (11) can be used to evaluate the tracking accuracy:

(11) T(k)=trace(P(k|k)).

## 4. The Optimal Sensor Scheduling Problem

### 4.1. System Assumptions

In our proposed algorithm, the assumptions made about the network model are as follows.

- The sensors and sink node are stationary.
- The sink node has strong computing ability and energy storage capacity with enough memory.
- The sink is aware of the locations of the sensor nodes.
- All sensor nodes are homogeneous (i.e., having the same sensing, processing, and communication capabilities).
- A sensor node and the sink node can communicate directly with each other via a single-hop link.

### 4.2. Target Tracking Mechanism

**Figure 1:**
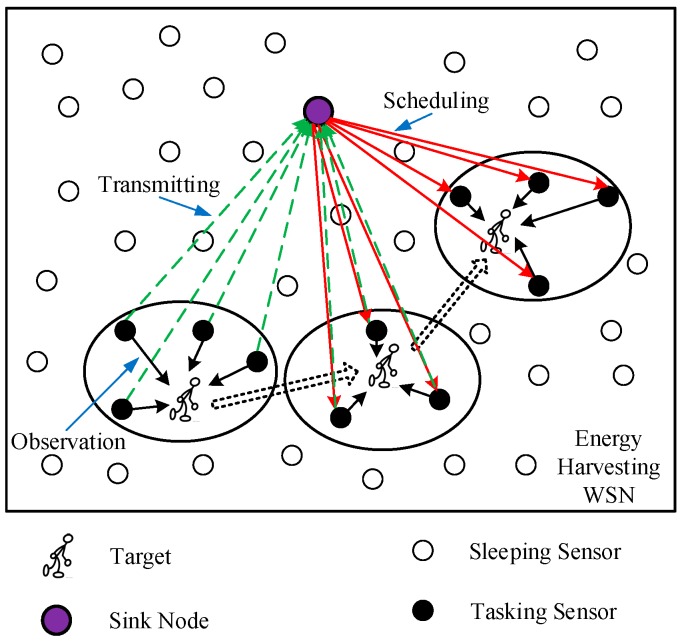
The target tracking system in an energy-harvesting wireless sensor network (WSN).

Figure 1 illustrates a target tracking scenario in an energy-harvesting WSN. When observed the target, the tasking sensors transmit the perceived measurements to the sink node. Then the sink node fuses the received measurements, predicts the target state for the next time step based on EKF, schedules the next tasking sensor nodes by ADP-MSS and notifies them by the low-power paging channel.

The general tracking system in the energy-harvesting WSN works as follows.

1. Initialization. When the target enters the sensor field, the energy-harvesting sensor with enough energy that detects the target for the first time becomes the first tasking sensor. It sends the measurement to the sink node.
2. State estimation and prediction. When the sink gets the new measurements, it estimates and predicts the state and error covariance by EKF.
3. Sensor scheduling. Based on the above solar energy harvesting model, the sink performs the sensor scheduling by ADP-MSS to minimize the performance which consists of the predicted tracking accuracy and energy consumption.
4. Mode swapping. The sink wakes up the tasking sensors for the current time step and switches the others to the sleeping mode via the low-power paging channel.
5. Monitoring and transmitting. The tasking sensors monitor the target and transmit the measurements to the sink.

### 4.3. Energy Consumption Analysis

At the *k*-th time step, the detection model of sensor *i* is described as

(12) $$D_{i}(k)=\left\{ \begin{array}{ll} 0 & E_{i}(k)<E^{h}\\ 1 & E_{i}(k)\geq E^{h} \end{array}\right.,$$

where $E^{h}$ is a threshold value for sensing the target, $E_{i}(k)$ represents the received signal level, and $E_{i}(k)=E_{i}^{0}\exp\left(-\beta d_{\left(x,i\right)}\right)$, in which $E_{i}^{0}$ and β are constant and $d_{\left(x,i\right)}$ is the Euclidean distance between the target and sensor i. The set of tasking sensors scheduled to track the target $\Omega_{T}(k)$ is a subset of $\Omega_{D}(k)=\{i|D_{i}(k)=1\}$, which denotes the set of all candidate sensors that possibly detect the target. At $t_{k}$, the energy consumption of sensor i is

(13) $$E_{con}(i)=\left\{ \begin{array}{ll} E_{r}+E_{t}(i)+E_{p} & u_{i}(k)=1\\ E_{s} & u_{i}(k)=0 \end{array}\right..$$

If $u_{i}(k)=1$, the sensor *i* is scheduled, otherwise the sensor *i* is sleeping. $E_{r}=e_{r}b_{r}$ represents the energy consumed to receive $b_{r}$ bits of data. $E_{t}(i)=(e_{t}+e_{d}d_{\left(s,i\right)}^{2})b_{t}$ represents the energy consumption due to transmitting $b_{t}$ bits of data to the sink node *s*. $E_{p}=e_{p}b_{p}$ represents the energy consumption due to sensing and data processing of $b_{p}$ bits, and $E_{s}$ represents the energy required for sleeping. $e_{r}$, $e_{t}$, $e_{d}$, and $e_{p}$ are decided by the specifications of the sensor.

In this paper, the design objective is to schedule the sensors for high tracking performance over an infinite horizon. Set the system state as the residual energy of the energy-harvesting sensors and the system control as the sensor scheduling solution. At $t_{k}$, before scheduled, the residual energy of sensor i is

(14) $$E_{i}^{bf}(k)=\min\left(E_{i}^{af}(k-1,u_{i}(k-1))+E_{h}(t_{k},\Delta t_{k}),H_{i}^{\max}\right)=f(E_{i}^{af}(k-1,u_{i}(k-1))),$$

where $E_{i}^{af}(k-1,u_{i}(k-1))$ is the residual energy of sensor i after being scheduled at the (*k* − 1)-th time step. If scheduled, sensor *i* must satisfy the restriction

(15) $$E_{i}^{bf}(k)\geq E_{r}+E_{t}(i)+E_{p}.$$

Let $\Omega_{u}(k)$ be the subset in $\Omega_{D}(k)$. For time step *k*, the scheduled sensors must be a subset of $\Omega_{u}(k)$. After being scheduled at the *k*-th time step, the consumed energy and residual energy of sensor *i* are

(16) $$E_{i}^{c}(k,u_{i}(k))=\left(E_{r}+E_{t}(i)+E_{p}\right)u_{i}(k)+E_{s}\left(1-u_{i}(k)\right),$$

(17) $$E_{i}^{af}(k,u_{i}(k))=E_{i}^{bf}(k)-E_{i}^{c}(k,u_{i}(k)).$$

According to (14), (15), and (17), we can obtain

(18) $$E_{i}^{af}(k,u_{i}(k))=f(E_{i}^{af}(k-1))+g_{i}u_{i}(k)-E_{s},$$

where $g_{i}=E_{s}-\left(E_{r}+E_{t}(i)+E_{p}\right)$. Let $g=\left[g_{1},g_{2},\cdots ,g_{M}\right]$, the system state of the *k*-th time step is $E_{af}(k)=\left[E_{1}^{af}\left(k,u_{1}(k)\right),E_{2}^{af}\left(k,u_{2}(k)\right),\cdots ,E_{M}^{af}\left(k,u_{M}(k)\right)\right]$, and the control of the *k*-th time step is $u(k)=\left[u_{1}(k),u_{2}(k),\cdots ,u_{M}(k)\right]$. Then, the system model is

(19) $$E_{af}\left(k\right)=f\left(E_{af}\left(k-1\right)\right)+g\times u(k)-E_{s},$$

where g×u(k) means the Hadamard product (i.e., element-wise product) between g and u(k).

## 5. ADP-Based Optimal Multi-Sensor Scheduling Algorithm

### 5.1. The Proposed Algorithm

We analyzed the predicted tracking accuracy and energy consumption respectively for time step *k*. To acquire the trade-offs between the potentially infinite network lifetime and the tracking accuracy, we define the utility function at time step *k* as

(20) $$U(k)=\beta_{1}T\left(k\right)+\sum_{i=1}^{M}E_{i}^{c}\left(k,u(k)\right),$$

in which $\beta_{1}>0$ is a coefficient to adjust the weight of the tracking accuracy [7]. It is obvious that U(k) is finite. Define the global performance index as the weight sum of the utility function from time step *k* to the infinite:

(21) $$J\left(k\right)=\sum_{j=k}^{\infty }\gamma^{j-k}U(j),$$

where 0<γ≤1 is a discount factor. Then, we can derive a Hamilton–Jacobi–Bellman (HJB) equation:

(22) J(k)=U(k)+γJ(k+1).

Hence, the objective function of the optimization multi-sensor scheduling problem for target tracking in an energy-harvesting WSN is

(23) $$\begin{array}{l} \;\underset{u(k)}{\min}\;J\left(k\right)\\ st\;\{\begin{array}{ll} D_{i}(k)=1 & \forall i\in \left\{i|u_{i}(k)=1\right\}\\ E_{af}(k)\geq 0 & \end{array} \end{array}$$

Let $J^{*}\left(k\right)=\underset{u(k)}{\min}J\left(k\right)$. Then, we can get the following HJB equation

(24) $$J^{*}\left(k\right)=\underset{u(k)}{\min}\left\{U\left(k\right)+\gamma J^{*}\left(k+1\right)\right\}$$

and the optimal control sequence $u^{*}(k)$ by

(25) $$u^{*}(k)=\arg\underset{u(k)}{\min}\left\{U\left(k\right)+\gamma J^{*}\left(k+1\right)\right\}$$

Generally, the optimal performance index function $J^{*}\left(k\right)$ is nonlinear, and it is difficult to obtain the optimal control by directly solving (24). To overcome the above problem, the ADP-MSS is proposed to get the approximate optimal solution in this paper.

**Figure 2:**
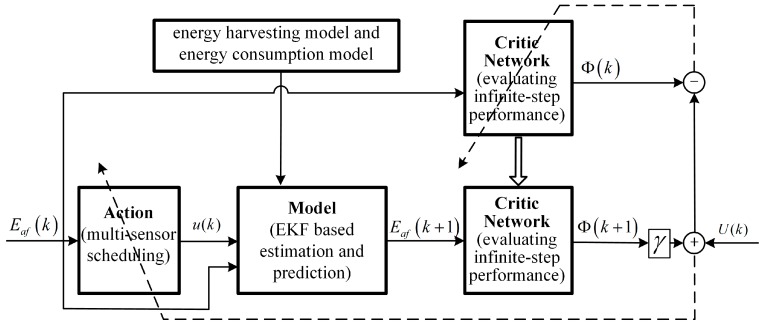
Structure of the adaptive dynamic programming based multi-sensor scheduling algorithm (ADP-MSS). EKF: extended Kalman filter.

A diagram of the proposed ADP-MSS is shown in Figure 2, which is comprised of three modules: model, critic network, and action. The model describes the relationship between the next system state $E_{af}(k+1)$ with the current system state $E_{af}(k)$ and the system control u(k) (i.e., the model in (19)). The critic network evaluating the infinite horizon performance is realized by a neural network, in which the input is the system state and the output is the evaluated performance index Φ(k) which tends to satisfy the HJB equation defined as in (22). The action is executed to find the optimal control for the evaluated performance in the critic network.

It runs as follows.

At first, let $\Phi^{\left[0\right]}(k)=0$ for any k, then we can obtain the optimal performance index at the first iteration step

(26) $$\Phi^{\left[1\right]}(k)=\underset{u(k)}{\min}\left\{U(k)+\gamma \Phi^{\left[0\right]}(k+1)\right\}$$

and the optimal control strategy

(27) $$u^{\left[0\right]}(k)=\arg\underset{u(k)}{\min}\left\{U(k)+\gamma \Phi^{\left[0\right]}(k+1)\right\}.$$

Next, when the iteration step *i* = 1, 2, ⋯, we can obtain

(28) $$\Phi^{\left[i+1\right]}(k)=\underset{u(k)}{\min}\left\{U(k)+\gamma \Phi^{\left[i\right]}(k+1)\right\},$$

(29) $$u^{\left[i\right]}(k)=\arg\underset{u(k)}{\min}\left\{U(k)+\gamma \Phi^{\left[i\right]}(k+1)\right\}.$$

The critic network is designed to approximate $\Phi^{\left[i+1\right]}$. The input is $E_{af}(k)\in R^{1\times M}$ where R is the set of real numbers and the output is

(30) $$\Phi^{\left[i+1\right]}(k)=w_{c}^{T}\left(k\right)\sigma \left(v_{c}^{T}\left(k\right)E_{af}\left(k\right)\right).$$

The optimal object can be expressed as

(31) $${\tilde{\Phi }}^{\left[i+1\right]}(k)=U(k)+\gamma \Phi^{\left[i\right]}(k+1).$$

Hence, we can define the error of the network as

(32) $$e_{c}^{\left[i+1\right]}(k)=\Phi^{\left[i+1\right]}(k)-{\tilde{\Phi }}^{\left[i+1\right]}(k).$$

Therefore, the objective function needed to be minimized in the critic network is $E_{c}^{\left[i+1\right]}={\left(e_{c}^{\left[i+1\right]}(k)\right)}^{2}/2$. The steepest descent method is used for the weight update:

(33) $$w_{c}^{\prime }(k)=w_{c}(k)-\alpha_{c}\partial E_{c}^{\left[i+1\right]}(k)/\partial w_{c}(k),$$

(34) $$v_{c}^{\prime }(k)=v_{c}(k)-\alpha_{c}\partial E_{c}^{\left[i+1\right]}(k)/\partial v_{c}(k),$$

in which $0<\alpha_{c}<1$ is the learning rate and the updated weights are $w_{c}^{\prime }(k)$ and $v_{c}^{\prime }(k)$.

### 5.2. The ADP-MSS Implementation Process

The pseudocode for ADP-MSS at time step *k* is given in Algorithm 1. Here, the system state $E_{af}\left(k\right)$ is known, δ is a very small positive value defined by the user, and $\Phi^{\left[i\right]}(k)$ denotes the iterative global performance index from time step *k* to the infinite, at iteration step *i.* This iteration procedure can be terminated after a predefined number of iteration step (*MI*) is reached.

**Algorithm 1** ADP-MSS1: set the value of $R_{i}$, q, $\beta_{1}$, γ, $\alpha_{c}$, X(1), P(1)2: select the initial value of $w_{c}\left(k\right)$, $v_{c}\left(k\right)$ randomly from a given region3: set *i* = 0; $\Phi^{\left[i\right]}(k)=0$, ∀k>0 and termination = false4: **while** (termination = false) **do**5:  $u^{\left[i\right]}\left(k\right)=\arg\underset{u(k)}{\min}\left\{U\left(k\right)+\gamma \Phi^{\left[i\right]}\left(k+1\right)\right\}$ (Action)6:  $E_{af}^{\left[i\right]}\left(k+1\right)=f\left(E_{af}\left(k\right)\right)+g\times u^{\left[i\right]}\left(k\right)-E_{s}$ (Model)7:  $\Phi^{\left[i\right]}\left(k+1\right)=w_{c}^{T}\left(k\right)\sigma \left(v_{c}^{T}\left(k\right)E_{af}^{\left[i\right]}\left(k+1\right)\right)$ (Critic Network)8:  ${\tilde{\Phi }}^{\left[i+1\right]}\left(k\right)=U\left(k\right)+\gamma \Phi^{\left[i\right]}\left(k+1\right)$ (HJB equation)9:  $\Phi^{\left[i+1\right]}\left(k\right)=w_{c}^{T}\left(k\right)\sigma \left(v_{c}^{T}\left(k\right)E_{af}\left(k\right)\right)$ (Critic Network)10:  $e_{c}^{\left[i+1\right]}\left(k\right)=\Phi^{\left[i+1\right]}\left(k\right)-{\tilde{\Phi }}^{\left[i+1\right]}\left(k\right)$11:  i=i+112:  **if**
$∥e_{c}^{\left[i+1\right]}\left(k\right)∥<\delta$ or i>MI
**then**13:   termination = true14:  **else**15:   updated the weight $w_{c}\left(k\right)$, $v_{c}\left(k\right)$ by the steepest descent method16:  **end if**17: **end while**18: return: $u^{\left[i\right]}\left(k\right)$

## 6. Performance Analysis

### 6.1. Theoretical Analysis

Now we will prove the convergence of ADP-MSS. That is, when i→∞, $\Phi^{\left[i\right]}(k)\to J^{*}(k)$.

**Theorem** **1.** *Let the proposed ADP-MSS be implemented according to (26)–(29), then* $\left\{\Phi^{\left[i\right]}(k),i=0,1,2,\cdots \right\}$ *is a bounded sequence.*

**Proof:**  Define a new sequence as follows:

(35) $$\Psi^{\left[i+1\right]}(k)=U^{\left[i\right]}(k)+\gamma \Psi^{\left[i\right]}(k),$$

in which $U^{\left[i\right]}(k)$ is the utility under the control $u^{\left[i\right]}(k)$, then $U^{\left[i\right]}(k)$ is bounded because the number of scheduled sensors is finite. Set $\Psi^{\left[0\right]}(k)=0$ for any k, and we can obtain

(36) $$\begin{array}{ll} \Psi^{\left[i+1\right]}(k) & =U^{\left[i\right]}(k)+\gamma \Psi^{\left[i\right]}(k+1)\\ & =U^{\left[i\right]}(k)+\gamma \left[U^{\left[i-1\right]}(k+1)+\gamma \Psi^{\left[i-1\right]}(k+2)\right]\\ & =U^{\left[i\right]}(k)+\gamma U^{\left[i-1\right]}(k+1)+\gamma^{2}\left[U^{\left[i-2\right]}(k+2)+\gamma \Psi^{\left[i-2\right]}(k+3)\right]\\ & =\cdots \\ & =\sum_{j=0}^{i}\gamma^{j}U^{\left[i-j\right]}(k+j)+\gamma^{i+1}\Psi^{\left[0\right]}(k+i+1)\\ & =\sum_{j=0}^{i}\gamma^{j}U^{\left[i-j\right]}(k+j) \end{array}$$

Hence, $\Psi^{\left[i+1\right]}(k)$ is bounded. According to (28), we can conclude that $\Phi^{\left[i+1\right]}(k)\leq \Psi^{\left[i+1\right]}(k)$, so $\left\{\Phi^{\left[i\right]}(k),i=0,1,2,\cdots \right\}$ is a bounded sequence. □

**Theorem** **2.** *Let the proposed ADP-MSS be implemented according to (26)–(29), then* $\left\{\Phi^{\left[i\right]}(k),i=0,1,2,\cdots \right\}$ *is a monotone non-decreasing sequence, that is,*

(37) $$\Phi^{\left[i+1\right]}(k)\geq \Phi^{\left[i\right]}(k).$$

**Proof:**  Mathematical induction is used in the proof.

At first, when i=0, $\forall k,\;\Phi^{\left[0\right]}(k)=0$. Then

(38) $$\Phi^{\left[1\right]}(k)\geq \Phi^{\left[0\right]}(k).$$

Assuming that $\Phi^{\left[l\right]}(k)\geq \Phi^{\left[l-1\right]}(k)$ for i=l−1, l=1,2,⋯, when i=l,

(39) $$\begin{array}{ll} \Phi^{\left[l+1\right]}(k) & =\underset{u(k)}{\min}\{U(k)+\gamma \Phi^{\left[l\right]}(k+1)\}\\ & \geq \underset{u(k)}{\min}\{U(k)+\gamma \Phi^{\left[l-1\right]}(k+1)\}\\ & =\Phi^{\left[l\right]}(k) \end{array}$$

Therefore, $\left\{\Phi^{\left[i\right]}(k),i=0,1,2,\cdots \right\}$ is a monotonic non-decreasing sequence. □

From Theorem 1 and Theorem 2, it can be inferred that $\left\{\Phi^{\left[i\right]}(k)\right\}$ is convergent. Denote

(40) $$\Phi^{\infty }(k)=\underset{i\to \infty }{\lim}\Phi^{\left[i\right]}(k).$$

**Theorem** **3.** *For any*k, $\Phi^{\infty }(k)$*is the optimal performance index, that is,*$\Phi^{\infty }(k)$*satisfies the HJB equation*

(41) $$\Phi^{\infty }(k)=\underset{u(k)}{\min}\{U(k)+\gamma \Phi^{\infty }(k+1)\}.$$

**Proof:**  From Theorem 2, we can get $\Phi^{\infty }(k)\geq \Phi^{\left[i+1\right]}(k)$. Let i→∞, then we can obtain

(42) $$\Phi^{\infty }(k)\geq \underset{u(k)}{\min}\{U(k)+\gamma \Phi^{\infty }(k+1)\}.$$

Based on the definition of $\Phi^{\infty }(k)$, ∀ε>0, $\exists \Phi^{\left[p\right]}(k)$, such that

(43) $$\Phi^{\left[p\right]}(k)\leq \Phi^{\infty }(k)\leq \Phi^{\left[p\right]}(k)+\varepsilon .$$

Then, we have

(44) $$\Phi^{\infty }(k)\leq \underset{u(k)}{\min}\{U(k)+\gamma \Phi^{\left[p-1\right]}(k+1)\}+\varepsilon .$$

ε can be ignored because it is any positive value. Let p→∞, then

(45) $$\Phi^{\infty }(k)\leq \underset{u(k)}{\min}\{U(k)+\gamma \Phi^{\left[\infty \right]}(k+1)\}.$$

From (42) and (45), we can get (41), which is just the definition of $J^{*}(k)$ after replacing $\Phi^{\infty }(\cdot )$ by $J^{*}(\cdot )$. Hence, we can conclude that $\Phi^{\infty }(k)=J^{*}(k)$, which means that the sequence of the iterative performance indexes in the proposed ADP-MSS will converge to the optimal solution. □

### 6.2. Simulation Results

**Figure 3:**
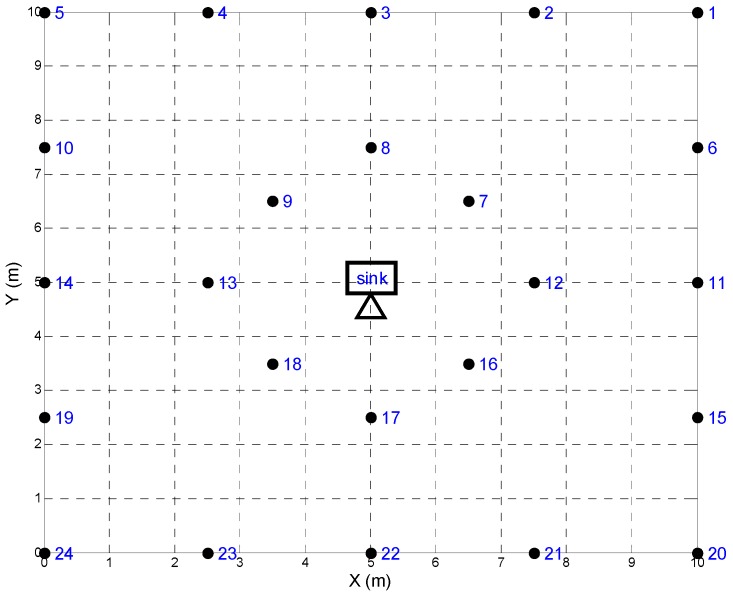
The layout of an energy-harvesting WSN.

In this paper we used Matlab 2014 as the simulation tool and considered a numerical example in which a WSN is deployed to monitor the moving target in a closed region with 10 m × 10 m square. The WSN contained 24 sensor nodes and one sink located at the center, as shown in Figure 3. For each sensor node, the sensing region was a circle centered on its own location with a radius of 3 m.

In the simulations of this paper, the ranging sensors were used to measure the distance between the sensor and the target. For sensor i located at $\left(x_{i},y_{i}\right)$, the measurement function $h_{i}$ is

(46) $$h_{i}\left(X(k)\right)=\sqrt{{\left(x(k)-x_{i}\right)}^{2}+{\left(y(k)-y_{i}\right)}^{2}}.$$

The Jacobian matrix for the measurement function is

(47) $$H(k+1)=\left[\begin{array}{cccc} \frac{x(k)-x_{1}}{\sqrt{{\left(x(k)-x_{1}\right)}^{2}+{\left(y(k)-y_{1}\right)}^{2}}} & 0 & \frac{y(k)-y_{1}}{\sqrt{{\left(x(k)-x_{1}\right)}^{2}+{\left(y(k)-y_{1}\right)}^{2}}} & 0\\ \vdots & \vdots & \vdots & \vdots \\ \frac{x(k)-x_{n}}{\sqrt{{\left(x(k)-x_{n}\right)}^{2}+{\left(y(k)-y_{n}\right)}^{2}}} & 0 & \frac{y(k)-y_{n}}{\sqrt{{\left(x(k)-x_{n}\right)}^{2}+{\left(y(k)-y_{n}\right)}^{2}}} & 0 \end{array}\right].$$

We assumed the solar panel’s area was 5 cm × 5 cm. The harvested energy rate was 0.1 W/cm^2^, and energy conversion efficiency was 15%. The max capacity of each battery was $5\times 10^{-2}$ J with the initial energy being $2.5\times 10^{-3}$ J, and they had infinite recharge cycles. Meanwhile, the variance of the measurement noise of the sensor nodes changed from 0.01 to 0.1. Except for $E_{s}$, the energy consumption parameters were borrowed from [31] as shown in Table 1, and the other constant parameters are given in Table 2.

In the simulations, the true trajectory of the target was a circle with a radius of 4 m centered at the center of the WSN. The residual energy of the 24 sensors at time step *k* $E_{af}(k)$ was used as the system state, and could be obtained by the previous system state estimation and the control according to (19). The control was the sensor scheduling scheme $u(k)=\left[u_{1}(k),u_{2}(k),\cdots ,u_{24}(k)\right]$, where $u_{i}(k)\in \left\{0,1\right\}$ and $u_{i}(k)=1$ means that sensor *i* was scheduled as one of the tasking sensors at time step *k*, otherwise the sensor *i* was not scheduled and could remain in the sleeping mode. While the target was moving in the monitoring area, the tracking system iteratively performed target detection by the scheduled tasking sensors, transmitting the measurements from the tasking sensors to the sink, target state estimation and prediction by the sink, and sensor scheduling by the sink. If the sensors are not properly scheduled, it can result in the failure of the tracking or degradation of the overall tracking performance.

**Figure 4:**
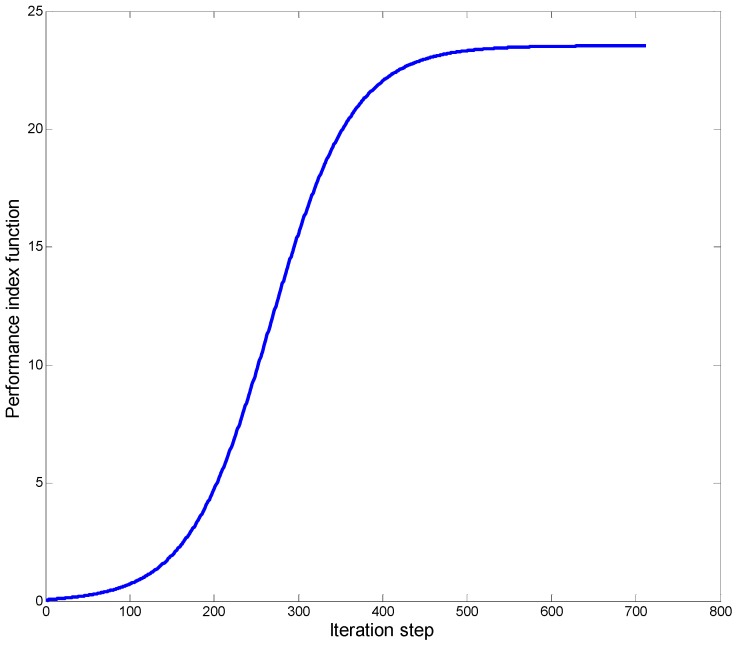
The changes of performance indexes with the iterations.

The structure of the adopted critic network was 24–30–1 with 24 inputs, 30 nodes in the hidden layer, and 1 output. Its initial weight values were set randomly from the range (0, 0.5). Figure 4 shows the changes of the performance indexes for the first time-step of ADP-MSS, initialized at 0. It can be found that the change of the performance indexes was monotone non-decreasing as analyzed in Theorem 2, and the curve converged after about 600 iterations.

**Figure 5:**
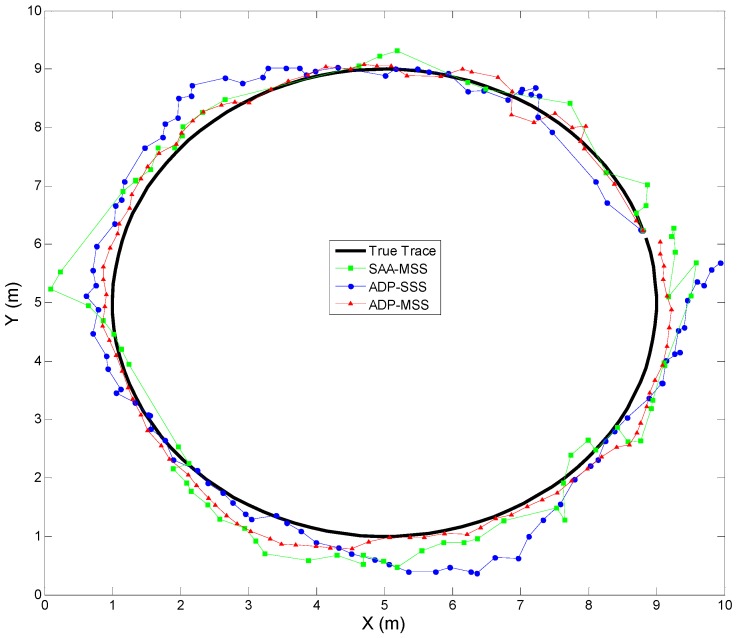
The true trace and estimated trajectories of the target when the variance of the measurement noise was 0.05 and the target speed was 5 m/s. ADP-MSS: ADP-based multi-sensor scheduling algorithm; ADP-SSS: ADP-based single-sensor scheduling algorithm; SAA-MSS: simulated annealing algorithm-based multi-sensor scheduling.

**Figure 6:**
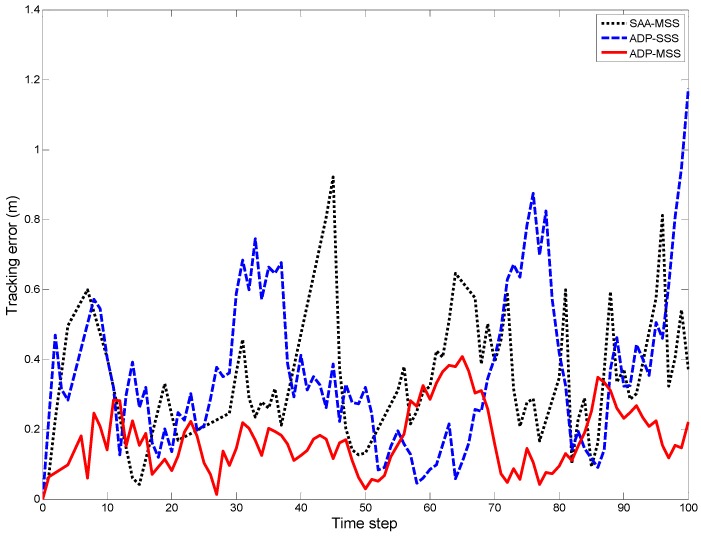
The tracking error when the variance of the measurement noise was 0.05 and the target speed was 5 m/s.

The true trace and estimated trajectories of the target are shown in Figure 5 when the variance of the measurement noise was 0.05 and the target speed was 5 m/s. The corresponding tracking error is shown in Figure 6, which consists of the Euclidean distance from the true coordinate to the estimated coordinate of the target at time step *k*. To evaluate the tracking accuracy of ADP-MSS, the tracking errors of an ADP-based single-sensor scheduling algorithm (ADP-SSS) and simulated annealing algorithm-based multi-sensor scheduling (SAA-MSS) are also shown in the same figure, where it is obvious that the proposed approach ADP-MSS was more stable and accurate.

**Figure 7:**
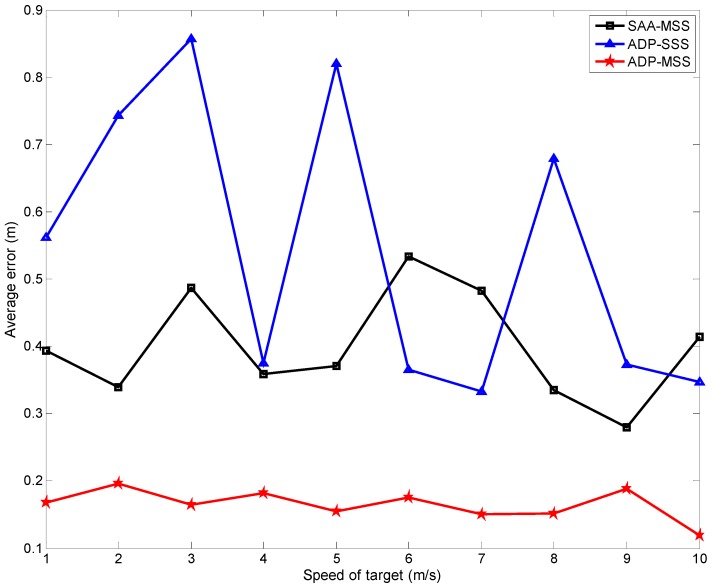
The average tracking error when the target speed changed from 1 m/s to 10 m/s.

**Figure 8:**
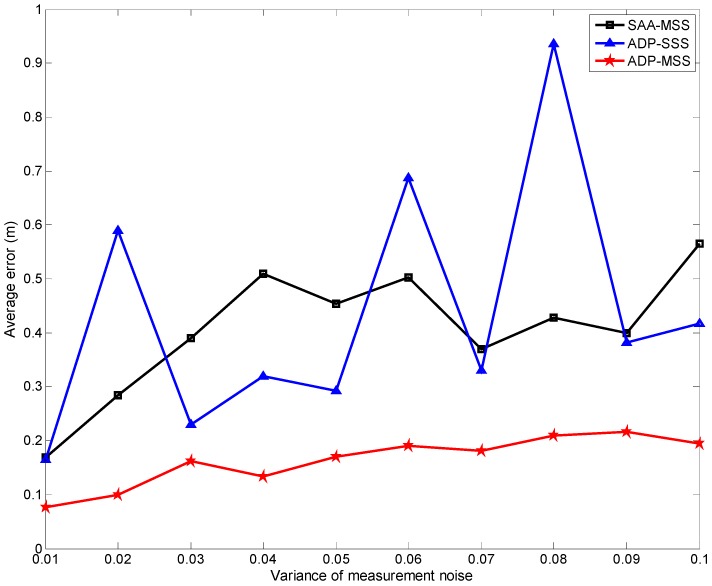
The average tracking error when the variance of the measurement noise changed from 0.01 to 0.1.

Figure 7 and Figure 8 show the tracking errors respectively while the target speed increased from 1 to 10 m/s and the variance of the measurement noise changed from 0.01 to 0.1. From the curves in these two figures, we can find that the results of the two multi-sensor scheduling schemes (ADP-MSS and SAA-MSS) were more stable and accurate than those of the single-sensor one (ADP-SSS). This is because multiple sensors can provide more information to improve the tracking accuracy using data fusion. In addition, it is easy to find that the results from ADP-MSS scheme were better than those from SAA-MSS. The main reason is that the SAA-MSS only takes the local optimization of the performance into account. In fact, the node’s state is associated with its previous state and may influence its states at the following steps. Hence, from the overall performance perspective, local optimal solutions are not the most reasonable decisions, and may have a negative impact on the global performance.

From Figure 4, Figure 5, Figure 6, Figure 7 and Figure 8, the following conclusions can be drawn:

- The performance index of ADP-MSS was monotonically non-decreasing and converged.
- The multi-sensor scheduling schemes were more stable and reliable than the single one.
- The proposed ADP-MSS could achieve global performance optimality.

## 7. Conclusions

ADP is an efficient method to solve the dynamic programming problems of discrete systems. This paper introduces the ADP approach (ADP-MSS) to the optimal multi-sensor scheduling problem for target tracking in energy-harvesting WSNs. We present an adaptive scheme to schedule the tasking sensors by considering the global optimization of the performance composed of the energy consumption and tracking accuracy over an infinite time horizon. Theoretical analysis proved that the iterative control by ADP-MSS will converge to the optimal solution. Through simulation results, we found that the multi-sensor scheduling schemes were more stable and reliable than the single sensor scheduling scheme and the proposed ADP-MSS was superior to an SAA-based multi-sensor scheduling scheme from a global perspective. As future work, more advanced ADP based cross-layer sensor network design schemes can be studied by jointly designing the network protocols with the sensor scheduling.

## Figures and Tables

**Table 1:** Parameters in the energy consumption model.

Parameters	Value
$e_{t}$	$4.5\times 10^{-5}\;J/\mathrm{bit}$
$e_{d}$	$1\times 10^{-11}\;J/\mathrm{bit}\cdot m^{2}$
$e_{r}$	$1.35\times 10^{-4}\;J/\mathrm{bit}$
$e_{p}$	$5\times 10^{-5}\;J/\mathrm{bit}$
$E_{s}$	$1\times 10^{-6}\;J$

**Table 2:** Simulation parameters.

Parameters	Value
process noise parameter *q*	1
coefficient $\beta_{1}$	0.10
discount factor γ	0.70
learning rate $\alpha_{c}$	0.20
sampling interval	0.05 s
Packet size in each transmission	10 bits
number of nodes in the NN hidden layer	30
initial location of the target	(8.81, 6.23)
computation precision δ	1 × 10^−3^
max iteration step *MI* in ADP algorithm	1000
